# Antimicrobial effect of blue light using *Porphyromonas gingivalis* pigment

**DOI:** 10.1038/s41598-017-05706-1

**Published:** 2017-07-12

**Authors:** Ayaka Yoshida, Haruka Sasaki, Toshizo Toyama, Mitsunori Araki, Jun Fujioka, Koichi Tsukiyama, Nobushiro Hamada, Fumihiko Yoshino

**Affiliations:** 1 Division of Photomedical Dentistry, Department of Oral Science, Graduate School of Dentistry, Kanagawa Dental University, 82 Inaoka-cho, Yokosuka, Kanagawa 238-8580 Japan; 2 Division of Microbiology, Department of Oral Science, Graduate School of Dentistry, Kanagawa Dental University, 82 Inaoka-cho, Yokosuka, Kanagawa 238-8580 Japan; 3 Department of Chemistry, Faculty of Science Division I, Tokyo University of Science, 1-3 Kagurazaka, Shinjuku-ku, Tokyo 162-8601 Japan

## Abstract

The development of antibiotics cannot keep up with the speed of resistance acquired by microorganisms. Recently, the development of antimicrobial photodynamic therapy (aPDT) has been a necessary antimicrobial strategy against antibiotic resistance. Among the wide variety of bacteria found in the oral flora, *Porphyromonas gingivalis* (*P*. *gingivalis*) is one of the etiological agents of periodontal disease. aPDT has been studied for periodontal disease, but has risks of cytotoxicity to normal stained tissue. In this study, we performed aPDT using protoporphyrin IX (PpIX), an intracellular pigment of *P*. *gingivalis*, without an external photosensitizer. We confirmed singlet oxygen generation by PpIX in a blue-light irradiation intensity-dependent manner. We discovered that blue-light irradiation on *P*. *gingivalis* is potentially bactericidal. The sterilization mechanism seems to be oxidative DNA damage in bacterial cells. Although it is said that no resistant bacteria will emerge using aPDT, the conventional method relies on an added photosensitizer dye. PpIX in *P*. *gingivalis* is used in energy production, so aPDT applied to PpIX of *P*. *gingivalis* should limit the appearance of resistant bacteria. This approach not only has potential as an effective treatment for new periodontal diseases, but also offers potential antibacterial treatment for multiple drug resistant bacteria.

## Introduction

Periodontal disease is a chronic inflammatory infection affecting the gingiva, and is associated with gingiva, periodontal ligament, and alveolar bone loss^1^. The etiology of periodontal disease as a bacterial infection is well established. Several subgingival bacteria including *Porphyromonas gingivalis* (*P*. *gingivalis*), *Prevotella intermedia*, *Tannerella forsythia*, *Aggregatibacter actinomycetemcomitans*, and spirochetes are leading candidates as etiologic agents in periodontal disease^2^. Some form of periodontal disease affects 75% of the population; severe forms affect 14% of adults of all ages and 30% of older adults^3^. Direct treatment costs due to dental diseases including periodontal disease worldwide were estimated at US$298 billion yearly, corresponding to an average of 4.6% of global health expenditure billion yearly^4^. *P*. *gingivalis* is one of the main pathogenic factors of common periodontal disease in adults and is widely recognized as the black pigment producing anaerobic gram-negative bacteria involved in the initial progression of periodontal disease^5–8^. Furthermore, the burden that *P*. *gingivalis* infections place on the body may be larger than previous estimates, as periodontal disease is also associated with an increased risk of systematic symptoms, such as coronary heart disease and diabetes^6, 9, 10^.

The first antimicrobial substance to be discovered was penicillin, and this marked the beginning of the “golden age of antibiotics.” Recently, a severe lack of control in the use of antibiotics, great abuse in areas such as livestock feed, and unnecessary prescriptions for viral infections have led to growing global rates of antibiotic resistance in microorganisms. The discovery of new antibiotics cannot keep up with the speed of resistance developed by microorganisms. The rise in antibiotic resistance worldwide has driven research into the development of new antibacterial strategies. However, overall, 74.2% of the patients with chronic periodontitis have been revealed subgingival periodontal pathogens resistant to at least one of the test antibiotics. Additionally, some of the periodontitis bacteria forming red complex included *P*. *gingivalis* have been reported to acquire resistance to the antibiotic such as amoxicillin. Amoxicillin resistance was present in 43.3% of the study patient which β-lactamase enzymes capable of hydrolyzing β-lactam antibiotics^11^. Many antibiotics for periodontal disease treatment was not indicated to be associated with the interacellular energy metabolism inhibition of oral bacteria. Therefore, it is very important to intracellularly target molecules and/or microorgans not related to antibiotic resistance mechanisms. For example, antimicrobial photodynamic therapy (aPDT) has the potential to be an alternative to antibiotics, especially for the treatment of localized skin infections^12^.

The first use of the photodynamic activity of chemical compounds and visible light against microorganisms was published at the beginning of the last century. Hermann von Tappeiner *et al*. reported that the observed toxic effect in the presence of the light was not attributed to heat. In 1904, von Tappeiner coined the term “photodynamic reaction” for the reaction of light with a non-toxic dye^13, 14^. After a long hiatus, in the 1970’s photo dynamic therapy (PDT) began to be explored for the selective destruction of malignancies^15^. Common features of tumor cells and microorganisms are high proliferation and active metabolisms. Therefore, if microorganisms can accumulate different photosensitizers, photodynamic inactivation of them might be effective^16^.

Even though the bactericidal properties of photodynamic effects have been known for a long time, only recently has there been increased interest in their practical use^17–20^. The development of resistance to aPDT appears to be unlikely because in microbial cells, reactive oxygen species (ROS), such as singlet oxygen (^1^O_2_) and some free radicals, interact with several cell structures and different metabolic pathways. Furthermore, aPDT is equally effective against antibiotic-resistant and antibiotic-susceptible bacteria, and repeated photosensitization has not induced the selection of resistant strains^21, 22^. There is also interest in applying aPDT against *P*. *gingivalis*, and studies have been conducted using visible light with wavelengths of 600 nm or more and with toluidine blue or methylene blue as a photosensitizer^18, 23, 24^. Visible light of 600 nm or greater is excellent for tissue penetration compared with shorter wavelengths due to low light absorption and scattering by living tissue^25^. Additionally, toluidine blue and methylene blue stain living tissues and cells as well as bacteria^26–28^. Therefore, these techniques using several stains may damage biological tissues and cells, even if it is effective as sterilization for *P*. *gingivalis*.

In this study, we focused on the dye porphyrin, which is the black pigment produced by *P*. *gingivalis*. First, we identified the excitation wavelength of porphyrin. Based on these results, we identified ROS induced by the photo-excited porphyrin in *P*. *gingivalis* and investigated the bactericidal effects from aPDT.

## Results

### Effect of blue light on PpIX fluorescence spectra

**Figure 1:**
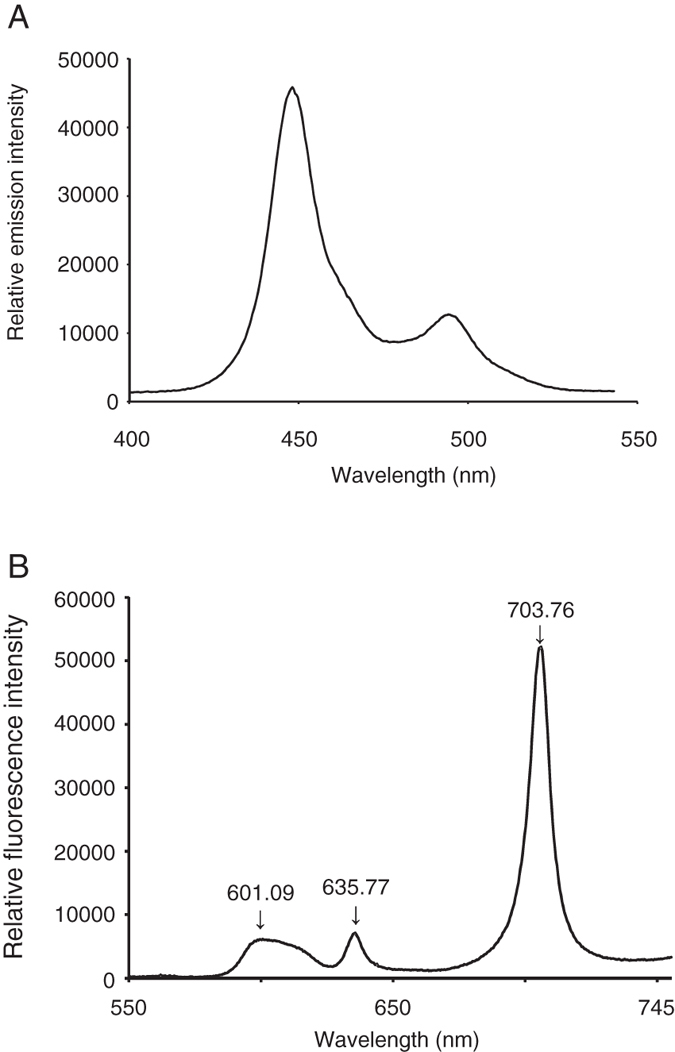
(**A**) Relative spectral emission curve from LED and (**B**) relative spectral fluorescence of PpIX with blue light irradiation.

The fluorescence spectrum of the blue LED light is shown in Fig. 1A. Following photolysis, the Protoporphyrin IX (PpIX) spectrum has three peaks: 601.09, 635.77, and 703.76 nm, and the highest peak is at 703.76 nm (Fig. 1B).

### ROS induced by blue light-irradiated PpIX

**Figure 2:**
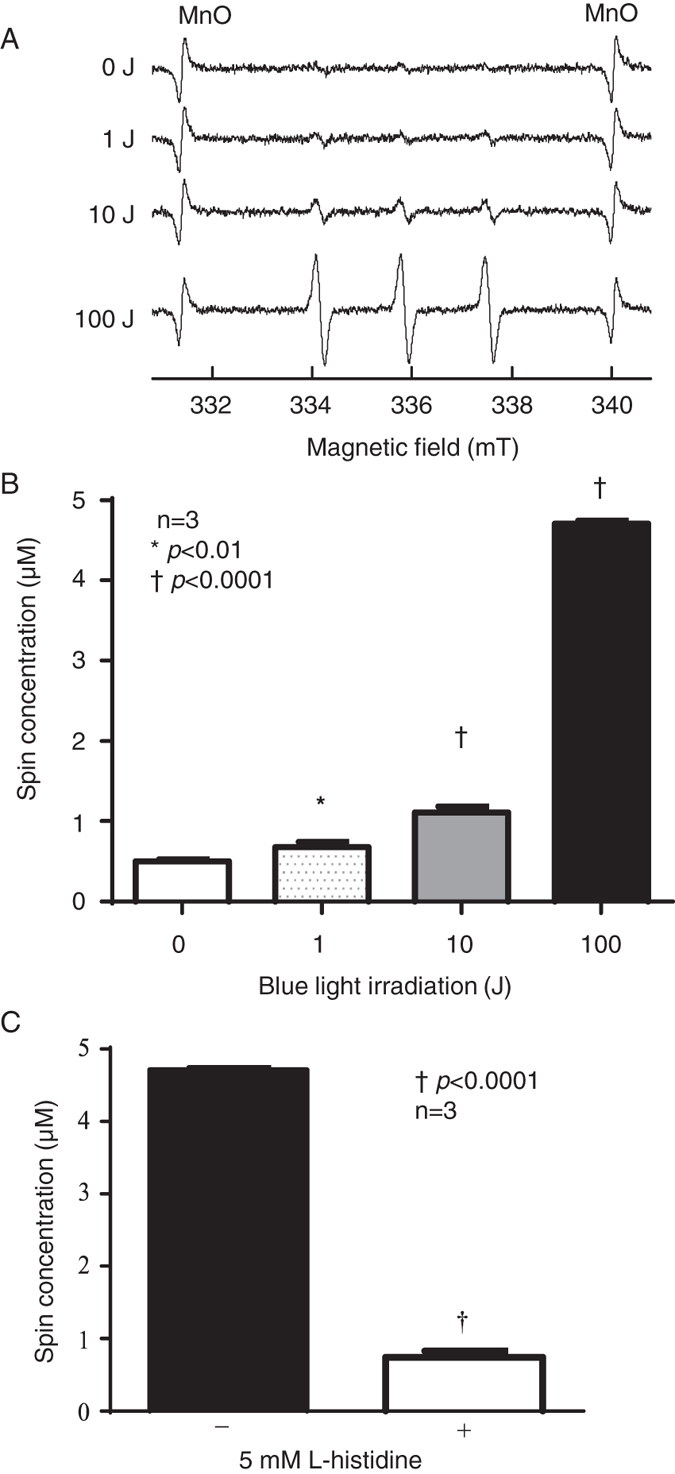
Singlet oxygen generation induced by PpIX upon blue light irradiation. (**A**) Typical *in vitro* ESR spectrum induced by PpIX upon blue light irradiation. (**B**) The concentration of generated singlet oxygen. (**C**) Generation of singlet oxygen with and without L-histidine at 100J of irradiation. The data are expressed as means ± SD (n = 3). Significant differences are expressed with * for *p* < 0.01 and † for *p* < 0.0001.

We investigated the ROS generated by PpIX upon blue light excitation. The characteristic ESR spectral pattern has three intense lines from the 2,2,6,6-tetramethyl-4-hydroxyl-piperidinyloxy (4-OH-TEMPO) radical, indicating that ^1^O_2_ generation was observed when using blue light to excite PpIX in the presence of 2,2,6,6-tetramethyl-4-piperidinol (4-OH-TEMP) (Fig. 2A). Even at high concentrations of PpIX, this generation of ^1^O_2_ was hardly observed without blue light irradiation. The spin concentration was about 5 µM with the 100J blue light irradiation of 100 µM PpIX (Fig. 2B). The spin concentration of products from 100 µM PpIX excited with 100J blue light was inhibited by the addition of L-histidine (Fig. 2C).

### Determination of PpIX contained in *P*. *gingivalis*

**Figure 3:**
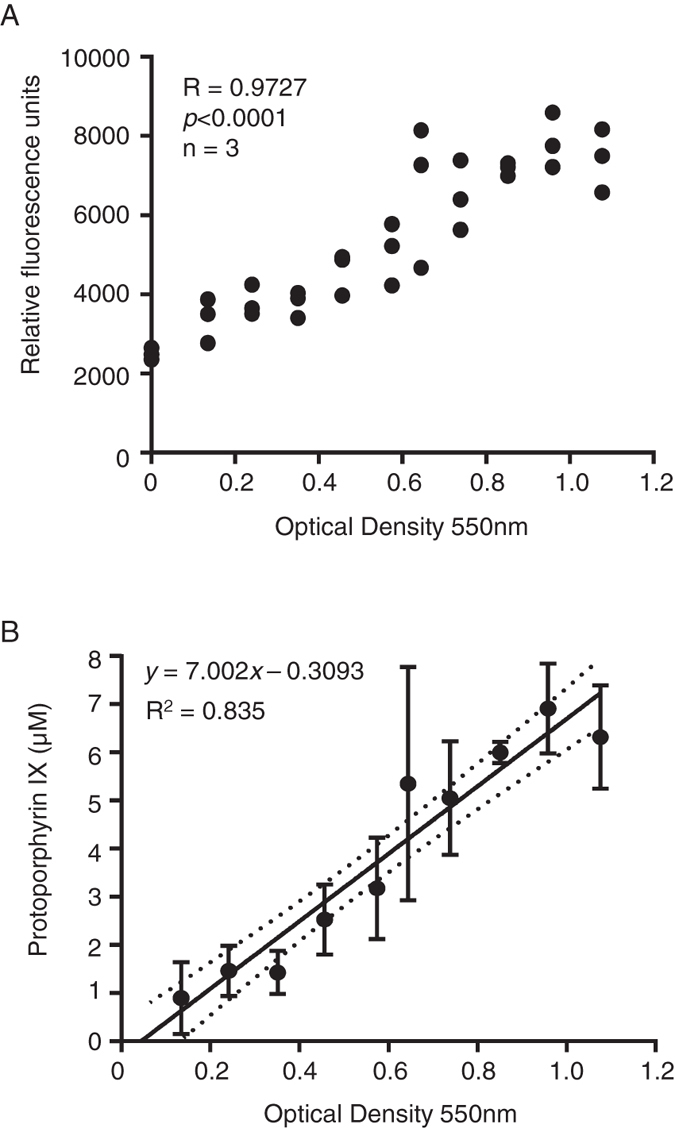
PpIX quantity in *P*. *gingivalis*. (**A**) Correlation of the relative fluorescence units and *P*. *gingivalis* concentration change (n = 3). (**B**) Linear regression between the concentration of *P*. *gingivalis* and PpIX (n = 3). Dots represent the 95% confidence curve.

We examined the importance of PpIX in *P*. *gingivalis* and the fluorescence wavelength of PpIX. The *P*. *gingivalis* suspension of each optical density (OD) was measured at the fluorescence wavelength (excitation 460 nm and emission 703 nm) of PpIX. We performed statistics using Spearman’s rank correlation coefficient, and there was a strong correlation (r = 0.9727, *p* < 0.001) between the OD of *P*. *gingivalis* and the relative fluorescence intensity (Fig. 3A). We converted the relative fluorescence intensity of each *P*. *gingivalis* concentration into a quantity of PpIX. Figure 3B shows the results of linear regression analysis after using Spearman’s rank correlation coefficient to prove the significant correlation between *P*. *gingivalis* concentration and PpIX level.

### Viability of *P*. *gingivalis* after blue light irradiation

**Figure 4:**
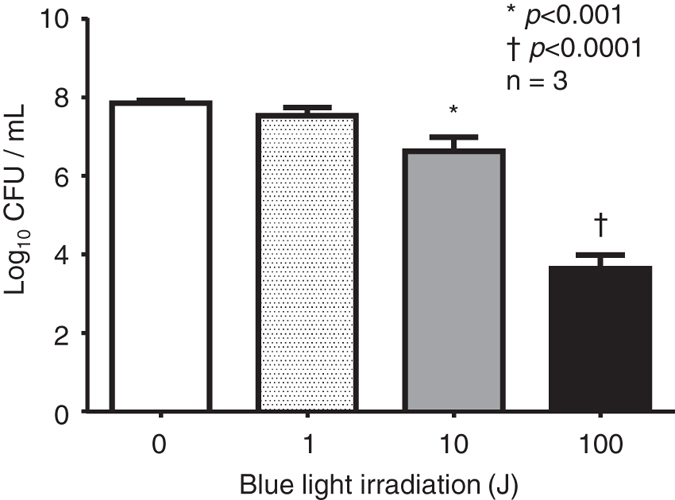
Effect of blue light irradiation on the viability of *P*. *gingivalis*. The data are expressed as means ± SD (n = 3). Significant differences are expressed with * for *p* < 0.001 and as † for *p* < 0.0001.

We investigated the effect of photodynamic therapy on *P*. *gingivalis* using blue light irradiation. As shown in Fig. 4, according to the logarithm of colony forming units (CFU) per mL, the viability of *P*. *gingivalis* was significantly suppressed in the 10J and 100J blue light irradiation groups compared with the non-irradiated (0J) group. However, no difference in survival was observed between the non-irradiated group and the 1J blue light irradiation group (Fig. 4).

### ROS production levels in *P*. *gingivalis* upon blue light irradiation

**Figure 5:**
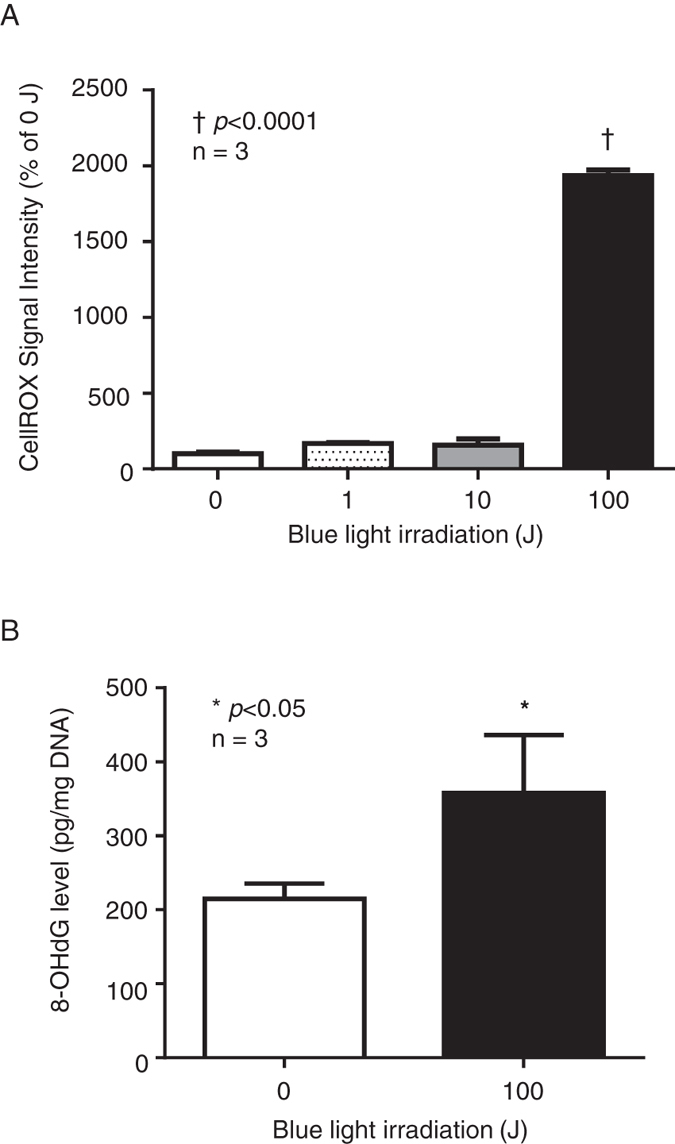
Oxidative stress induced by blue light irradiation of *P*. *gingivalis*. (**A**) ROS generation levels upon blue light irradiation in *P*. *gingivalis*. (n = 3) ^†^Significant difference (*p* < 0.0001). (**B**) Generation of 8-OHdG upon blue light irradiation of *P*. *gingivalis* (n = 3) *Significant difference (*p* < 0.05).

We examined ROS generation induced by blue light irradiation in *P*. *gingivalis*. The *P*. *gingivalis* suspension containing 20 µM CellROX ^®^ Green Reagent was irradiated with blue light. While ROS levels were significantly higher in the 100J blue light irradiation group compared with the non-irradiated (0J) group, there was no difference between the 0, 1, and 10J blue light irradiation groups (Fig. 5A). As shown in Fig. 5B, the oxidative stress levels of *P*. *gingivalis* DNA at 100J of blue light irradiation had significantly higher 8-OHdG levels compared with the non-irradiated (0J) group.

## Discussion

In recent years, because of the worldwide increase in antibiotic resistant bacteria, studies have been conducted on antimicrobial therapies using photodynamic therapy, which was already being used in cancer treatment^29^. Among the wide variety of bacteria in the oral cavity flora, the bacteria of periodontal disease are used for the study and application of aPDT in clinical practice. However, the current photodynamic therapy method is complicated, as it includes staining with a photosensitizer, and the bacterial sterilization mechanism has not been clarified^18, 23, 24^. In this study, we examined the sterilization mechanism of aPDT in bacterial cells using PpIX, a pigment used for energy production by *P*. *gingivalis*.

*P*. *gingivalis* requires iron and PpIX for growth^30–32^. The absorption spectrum of PpIX includes a maximal peak at 410 nm (Soret band) and four smaller peaks (Q-bands) from 500 to 630 nm^33^. Blue light at wavelengths of 400–500 nm is used for oral treatments such as resin restoration and tooth whitening^34–36^. The fluorescence wavelength peak (Fig. 1B) of PpIX excited with blue light (Fig. 1A) was consistent with the fluorescence wavelength for photodynamic diagnoses used in cancer therapy (600–740 nm)^37, 38^.

We attempted to detect ROS generated upon blue light irradiation of PpIX using an ESR technique used specifically for detecting ROS. Blue light irradiation of PpIX generates ^1^O_2_, a critical ROS (Fig. 2A,B). The inhibition of this ROS by typical scavenger L-histidine confirms it as ^1^O_2_^39, 40^ (Fig. 2C). ^1^O_2_ is generated by photochemical reactions through the transfer of excitation energy from a suitable triplet state sensitizer or by radical interactions^41^. Furthermore, ^1^O_2_ is a powerful oxidizing molecule, and starts further oxidation reactions in closed environments such as bacterial cell walls, lipid membranes, enzymes, or nucleic acids^42–44^. ^1^O_2_ generated upon the blue light irradiation of PpIX increased in proportion to irradiation intensity (Fig. 2A,B).

We examined the presence of PpIX in *P*. *gingivalis* to investigate the possibility of ^1^O_2_ generation. The fluorescence intensity of *P*. *gingivalis* was measured using the excitation (460 nm) and emission (703 nm) wavelengths of PpIX, and the concentration of *P*. *gingivalis* and the relative fluorescence intensity increased proportionally, with a strong correlation (Fig. 3A). The observed correlation with the relative fluorescence intensity of *P*. *gingivalis* (Fig. 3B) suggested the presence of a photosensitizer like PpIX in *P*. *gingivalis* cells. Due to the presence of the photosensitizer-like pigment, we chose to investigate the effects of blue light irradiation on *P*. *gingivalis*. A decrease in the viability of *P*. *gingivalis* was observed with 10 and 100J of blue light irradiation (Fig. 4).

**Figure 6:**
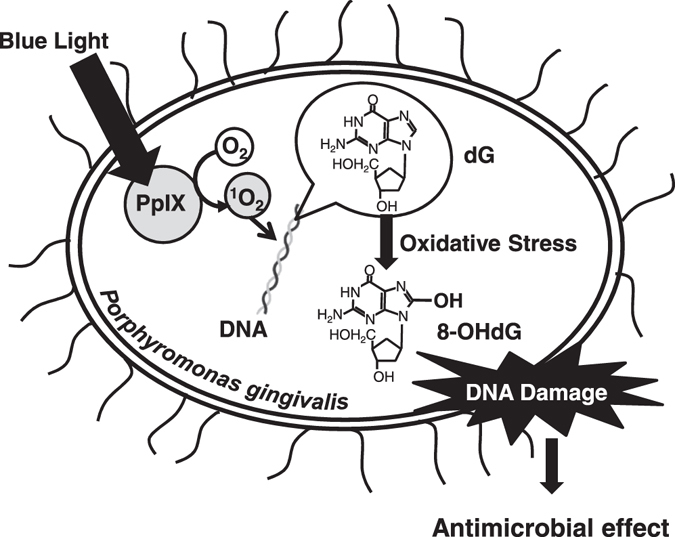
Blue light irradiation-induced antimicrobial mechanisms via protoporphyrin IX (PpIX) in *Porphyromonas gingivalis*.

CellROX® Green Reagent is a cell-permeable reagent used to stain DNA that emits fluorescence in response to oxidative stress. This reagent can stain and be measured in Gram-negative bacteria such as *Escherichia coli* as well as in mammalian cells^35, 45^. The same irradiation protocol and viability measurements were performed with this reagent, and oxidative stress increased during 100J of irradiation (Fig. 5A). 8-OHdG is a constituent DNA base, deoxyguanosine (dG), hydroxylated at the C-8 position, and is a DNA oxidative damage marker. Since dG has the lowest redox potential of the four bases of DNA, it is susceptible to oxidation by ROS. For this reason, 8-OHdG, the major oxidation product of dG, sensitively reflects the impact of ROS on the organism^46, 47^. We evaluated 8-OHdG and observed significant enhancement in the 100J irradiation group in comparison with the 0J group (Fig. 5B). There was no effect of DNA oxidative damage with 10J blue light irradiation. We considered that the cell membrane was influenced the oxidative damage by low power irradiation. However, differences in lipid peroxidation, which is another possible way to evaluate oxidative stress in the cell membrane, was not detected (data not shown). Therefore, the mechanism is still unclear that ROS induced by 10J blue light irradiation suppressed CFUs. Nevertheless, ^1^O_2_ was generated by the intracellular pigment-like PpIX of *P*. *gingivalis* upon blue light irradiation, which then directly causes oxidative damage to DNA, inhibiting bacterial growth. Collectively, it was found that it might be possible to sterilize *P*. *gingivalis* using only the intracellular pigment-like PpIX and blue light irradiation (Fig. 6).

This approach is not a traditional aPDT approach, which is to use an exogenous photosensitizer such as toluidine or methylene blue to stain the bacteria. It is a simple method using a *P*. *gingivalis*-specific pigment. In addition, there is no possibility for the appearance of resistant bacteria in the future due to the photosensitizer being in the bacterium itself. Therefore, this method might be revolutionary and could change periodontal therapies using various other antimicrobial agents. However, phototoxicity is an issue that with enough evidence to cause uneasiness with this method, as blue light in itself brings about oxidation stress in oral tissues^34^. On the other hand, we have already studied blue light irradiation and cytotoxicity within the oral tissues, and determined that taking antioxidants such as *N*-acetyl-L-cysteine could defend against cytotoxicity, since the antioxidant is maintained in vascular circulation. The antioxidant remains in the blood circulation of the host body and provides the cells and tissues with protection while the oral bacteria remain susceptible with blue light irradiation^34–36, 39^. Therefore, the antioxidant effect of NAC is not applied to bacteria, applying the NAC might protect the host body tissue and be possible to directly damage a *P*. *gingivalis* bactericidal activity by blue light irradiation.

In summary, our approach might be able to inspire new periodontal disease treatment only targeting the performance *P*. *gingivalis*, while taking into consideration the application of antioxidants before aPDT for protection of the oral tissues. In addition, aPDT using several exogenous dye has already been performed and clinical research results have been reported on periodontal disease treatment^18, 23, 24^. Unfortunately, this present study as aPDT which does not use exogenous dye, has not been carried out *in vivo* human study. We are going to need to implement in future dental aPDT treatment for the establishment of novel periodontal treatment.

## Materials and Methods

### Reagents

4-hydroxy-2,2,6,6-tetramethylpiperidine-*N*-oxide (TEMPOL), 4-OH-TEMP and PpIX disodium were purchased from Sigma-Aldrich (St. Louis, MO, USA). Phosphate buffered saline pH 7.2 (PBS) was purchased from the Invitrogen Corporation (Carlsbad, CA, USA), and L-histidine was purchased from Wako Pure Chemical Industries, Ltd. (Osaka, Japan). All other reagents were of analytical grade.

### Lighting source and conditions

Techno Light KTL-100 (Light emitting diode; LED), with a mounted light guide tip diameter of 4.3 mm and a blue transmission filter (225S-SPF500), were purchased from the Kenko Tokina Corporation (Tokyo, Japan). The LED output power was set to 400 mW/cm^2^ and wavelength to 460 nm using an optical power meter (8230E, ADC Corporation, Tokyo, Japan) before each experiment. *P*. *gingivalis* suspensions in each well were irradiated with the light source tip from the surface edge of the plate top at a depth of 10.9 mm for 96-well plates (Clear bottom black plate, Corning Incorporated, Corning, NY, USA) or 14.6 mm for 24-well plates (Clear bottom black plate, Eppendorf AG, Hamburg, Germany).

### Spectroscopic Analysis

The fluorescence spectra of 1 mM PpIX irradiated by blue light were obtained with an Exemplar (BRC115P, B&W TEK, Newark, DE, USA) connected to BWSpec 4 (B&W TEK, Newark, DE, USA).

### *In vitro* electron spin resonance (ESR) measurement

^1^O_2_ generated upon blue light irradiation of PpIX was analyzed quantitatively using ESR spectroscopy^48, 49^. It was detected by adding ^1^O_2_ trapping agent (100 mM 4-OH-TEMP) to PpIX solutions irradiated with blue light (460 nm, 400 mW/cm^2^). The concentration of the PpIX solution was 100 µM and blue light irradiation doses were 0, 1, 10, and 100J. We assessed the inhibitory effect of ROS in blue light irradiated PpIX solutions with 5 mM L-histidine^34^. We compared the double integrals of 4-OH-TEMP experimental spectra with those of a 10 μM TEMPOL standard measured under identical settings to estimate ^1^O_2_ adduct concentration^50^. ESR was performed using a JES-RE1X (JEOL, Tokyo, Japan) connected to a WIN-RAD ESR Data Analyzer (Radical Research, Tokyo, Japan) at the following instrument settings: microwave power, 8.00 mW; magnetic field, 335.8 ± 5.0 mT; field modulation width, 0.1 mT; sweep time, 1 min; and time constant, 0.03 s. All experiments were repeated three times.

### Bacterial strain and cultivation conditions

The bacterial strain used in this study was *P*. *gingivalis* ATCC 33277. *P*. *gingivalis* was grown in brain heart infusion broth (BHI broth; Difco Laboratories, Detroit, MI, USA) supplemented with 5 mg/ml yeast extract, 5 μg/ml hemin and 1 μg/ml vitamin K_1_ (BHIY-HK broth) for 18 h. *P*. *gingivalis* was also grown on BHI blood agar medium containing defibrinated 5% sheep blood at 37 °C for 5 days under anaerobic conditions (85% N_2_, 10% H_2_, 5% CO_2_). *P*. *gingivalis* was washed twice with PBS before each experiment to avoid the influence of medium pigments.

### Fluorescence analysis of *P*. *gingivalis*

We adjusted each of the washed *P*. *gingivalis* suspensions to an optical density of 550 nm (OD_550_; 0, 0.1, 0.2, 0.3, 0.4, 0.5, 0.6, 0.7, 0.8, 0.9, and 1.0) and then placed 150 μL per well into a 96-well plate. We measured the fluorescence of each well with infiniteM200 (Tecan Group Ltd., Männedorf, Switzerland) connected to PLATEmanagerV5 (Wako Pure Chemical Industries, Ltd, Osaka, Japan). The optimal excitation wavelength for detection was 460 nm and the emission wavelength was 703 nm.

### Survival of *P*. *gingivalis* after blue light irradiation

The washed *P*. *gingivalis* suspensions were adjusted to an optical density of 0.6 at 550 nm and added to a 96-well plate at 150 µL per well, and then irradiated with 0, 1, 10, or 100J (1J; 400 mW/cm^2^, 2.5 sec. 10J; 400 mW/cm^2^, 25 sec. 100J; 400 mW/cm^2^, 250 sec). The survival of the *P*. *gingivalis* cells was estimated from viable bacteria by counting the number of CFUs 5 days after culture on BHI agar media under anaerobic conditions. The data is shown as the logarithm of CFU per milliliter.

### ROS production and oxidative stress assay

The ROS production levels in blue light-irradiated *P*. *gingivalis* were measured with CellROX^®^ Green Reagent (Promega Corporation, Madison, WI, USA). The washed *P*. *gingivalis* was adjusted to an optical density of 0.6 at 550 nm. *P*. *gingivalis* was suspended in PBS with 20 µM CellROX^®^ Green Reagent and placed into a 96-well plate at 150 µL per well and irradiated for 0, 1, 10, or 100J. Non-target wells were covered with aluminum foil. After irradiation, the entire 96-well plate was covered with aluminum foil and incubated for 30 min at room temperature. The fluorescence intensity was measured with an infiniteM200 (Tecan Group Ltd., Männedorf, Switzerland) connected to PLATEmanagerV5 (Wako Pure Chemical Industries, Ltd., Osaka, Japan) at an excitation wavelength of 485 nm and an emission wavelength of 520 nm.

We also examined the oxidative stress on *P*. *gingivalis* DNA. The washed *P*. *gingivalis* was adjusted to an optical density of 0.6 at 550 nm and 1.5 mL per well was placed in a 24-well plate and irradiated at 0 or 100J. The precipitates obtained from collected samples after 2 min centrifugation at 12000 G were used for DNA extraction using ISOPLANT, according to the manual (Nippon Gene Co., LTD., Toyama, Japan). After measuring the DNA concentration with the NanoDrop ND-1000 spectrophotometer (NanoDrop Technologies, Inc., Wilmington, DE, USA) connected to NanoDrop 1000 Version 3.8.0 (Thermo Fisher Scientific, Inc., Waltham, MA USA), DNA was prepared with an 8-hydroxy-2′-deoxyguanosine (8-OHdG) Assay Preparation Reagent Set according to the manual (Wako Pure Chemical Industries, Ltd., Osaka, Japan). 8-OHdG was measured with a DNA/RNA Oxidative Damage ELISA kit, following the manufacturer’s instructions (Cayman Chemical Company, Ann Arbor, MI). After incubation for 90 min at room temperature, the absorbance was measured at 412 nm. The concentration of 8-OHdG was normalized to DNA concentration.

### Statistics

Data are represented by the mean ± standard deviation of minimum for the three separate experiments. All statistical analyses were performed with unpaired t-tests or Tukey’s multiple comparisons tests using GraphPad Prism 6 software (GraphPad Software Inc., La Jolla, CA, USA). A P-value of less than 0.05 was considered statistically significant.
